# Author Correction: On the evolution of sexual receptivity in female primates

**DOI:** 10.1038/s41598-020-72316-9

**Published:** 2020-09-04

**Authors:** Kelly Rooker, Sergey Gavrilets

**Affiliations:** 1 Department of Mathematics, University of Tennessee, Knoxville, TN 37996 USA; 2 Department of Ecology and Evolutionary Biology, University of Tennessee, Knoxville, TN 37996 USA; 3 National Institute for Mathematical and Biological Synthesis, University of Tennessee, Knoxville, TN 37996 USA; 4 Center for the Dynamics of Social Complexity, University of Tennessee, Knoxville, TN 37996 USA

Correction to: *Scientific Reports* 10.1038/s41598-020-68338-y, published on 20 July 2020

The Acknowledgements section in this Article is incomplete.

“We thank S. Perry, S. Hrdy, and reviewers for comments and suggestions. K.R. was supported by a National Science Foundation Graduate Research Fellowship. S.G. was supported by the National Institute for Mathematical and Biological Synthesis through National Science Foundation awards EF-0832858 and DBI-1300426, with additional support from The University of Tennessee.”

should read:

“We thank S. Perry, S. Hrdy, and reviewers for comments and suggestions. K.R. was supported by a National Science Foundation Graduate Research Fellowship. S.G. was supported by the National Institute for Mathematical and Biological Synthesis through National Science Foundation awards EF-0832858 and DBI-1300426, with additional support from The University of Tennessee. Funding for open access to this research was provided by University of Tennessee’s Open Publishing Support Fund.”

