# Supplementary material for: On the evolution of sexual receptivity in female primates
Source: Sci Rep. 2020 Jul 20;10:11945. doi: 10.1038/s41598-020-68338-y (PMC7371874; doi:10.1038/s41598-020-68338-y)
Supplement: Supplementary file 1 — Supplementary information. [file 41598_2020_68338_MOESM1_ESM.pdf]

Supplementary Information for:

On the evolution of sexual receptivity in female primates

Kelly Rooker and Sergey Gavrilets

Contents

A Overview, Design Concepts, and Details (ODD) Protocol for Individual-Based Computer Simulations **2**

A.1 Overview . . . 2

A.1.1 Purpose . . . 2

A.1.2 Entities, State Variables, and Scales . . . 2

A.1.3 Process Overview and Scheduling . . . 5

A.2 Design Concepts . . . 5

A.2.1 Emergence . . . 5

A.2.2 Adaptation . . . 7

A.2.3 Fitness . . . 7

A.2.4 Learning and Prediction . . . 8

A.2.5 Sensing . . . 8

A.2.6 Interaction . . . 8

A.2.7 Stochasticity . . . 9

A.2.8 Collectives . . . 9

A.2.9 Observation . . . 9

A.3 Details . . . 9

A.3.1 Initialization . . . 9

A.3.2 Input Data . . . 10

A.3.3 Submodels . . . 10

B List of all Parameters **11**

C Effects of all Parameters **14**

C.1 ANOVA Results 16

C.2 Supplementary Figures 17

A Overview, Design Concepts, and Details (ODD) Protocol for Individual-Based Computer Simulations

Here we list further details of the model and implementation, in the ODD protocol format (Grimm, V et al. "The ODD protocol for describing agent-based and other simulation models: A second update to improve clarity, replication, and structural realism." *Journal of Artificial Societies and Social Simulation* **2**, 7 (2020).

A.1 Overview

A.1.1 Purpose

The purpose of the model in this article is to propose a general framework for modeling the evolution of sexual receptivity in female primates. Using this model, we will investigate the following questions: How does length of sexual receptivity contribute to female reproductive success? What factors facilitate the evolution of continuous receptivity and/or very short lengths of receptivity? What role could infanticide have in the evolution of receptivity? How is receptivity linked to the evolution of sexual attractiveness, namely a female having obvious visual ovulation signs or concealed ovulation?

A.1.2 Entities, State Variables, and Scales

We consider a population of individuals interacting in G groups, each comprised of N males and N females. Females can differ genetically in both their visible ovulation signs present (with

magnitude m and length ℓ) and their length of receptivity (r), while males differ in their quality to females, meaning any benefit from the male to a female and/or her offspring. We do not consider evolution in males, assuming instead that male traits are at a [stochastic] evolutionary equilibrium.

Male-provided genetic (y_g) and non-genetic (y_{ng}) benefits are randomly drawn from the bivariate normal distribution, each with mean $\hat{y}$ and standard deviation b, and correlation parameter ρ; parameter $\hat{y}$ characterizes mean male quality and $b > 0$ the extent of variation. Males are ranked according to the value of their genetic quality: $y_{g,j}$ such that $y_{g,1} > y_{g,2} > ...$ for each male j. Small b implies small additional benefits to females from investing in ovulation signaling.

Our model explicitly accounts for the female cycle by using D discrete units of time. Without loss of generality, we refer to these units of time as 'days' (e.g., $D = 29$ days). For each day of the cycle, every female will have an associated probability of fertilization. We assume each fertile period to last $C \leq D$ days (e.g., $C = 7$). For all days lying outside these C fertile days, females are assumed to have zero probability of fertilization but can still mate if receptive on those days. We assume a triangular shape for the fertility function to be identical in all females, but with midpoints randomly distributed so as cycle synchrony occurs only probabilistically. Visual ovulation signs and receptivity are both correlated with these days of fertility (in that a female's day of peak ovulation signs will align with both her median day of receptivity and her peak day of fertility), but can each still evolve freely.

Although the form of such fertile periods will be identical in all females, the timing of each fertile period will be randomly distributed among females. For instance, when $C = 7$ (calling each of these fertile days $C1$, $C2$, ... , $C7$) and $D = 29$ (calling each of these days $D1$, $D2$, ... , $D29$), $C1$ in every female has an equal probability of landing on any of $D1$, $D2$, ... , $D29$. Since C is a cycle, we similarly assume $C7$ can land on any such day. For example, if $C1$ landed on $D24$, then $C7$ would 'wrap around' the cycle to land on $D1$. Moreover, for all females we assume ovulation happens directly in the middle of this C cycle. Hence, for $C = 7$ we assume ovulation happens on $C4$ and the probabilities of fertilization increase from $C1$ to $C4$ and then decrease from $C4$ to

C7.

Each female i will have $r_i \leq D$ days of receptivity. These days of receptivity line up with the female's days of fertility, such that a female's median day of fertility will equal her median day of receptivity. Similarly, since having visible ovulation signs at least loosely correlates with a female's fertility, we assume the day(s) of having maximum visual ovulation signs also align with the day(s) of that female's maximum fertilization probability, and align these cycles accordingly (note in most cases, $C \neq r_i \neq \ell_i$).

Let r be the number of days of the cycle a female is receptive to mating (a non-negative, integer value). We treat visual ovulation signs, $x(d)$ for each day d of the cycle, as overlapping graded curves. Each female's curve is characterized by two traits: magnitude (m) and length (ℓ). m is the maximum amount of ovulation signs a female has visible during her cycle (a non-negative, continuous value), while ℓ is the number of days a female has *some* amount of ovulation signs visible (a non-negative, integer value).

Given female traits $m_i \geq 0$ (ovulation signs magnitude) and $\ell_i \in \mathbb{Z}, \ell_i \in [0, D]$ (ovulation signs length), the amount $x_i(d)$ of ovulation signs visible on any day d of the cycle is defined as

$$x_i(d) = \begin{cases} m_i \cdot \left[1 - \left(\frac{2|d - \lceil \ell_i/2 \rceil|}{\ell_i + 1}\right)^{\gamma}\right] & \text{for } d = 1, 2, ..., \ell_i \\ 0 & \text{otherwise} \end{cases} \tag{S1}$$

for parameter $\gamma > 0$ and d measured from the start of ovulation signs being visible. Mutation effects in m_i are randomly chosen from the normal distribution $N(0, \sigma^2)$, whereas mutations in ℓ_i and r_i are discrete, representing adding or subtracting exactly one day to or from a female's time of having ovulation signs visible or being receptive, respectively.

Note that the exact function $x_i(d)$ is chosen such that (i) m_i will denote the peak of the curve, (ii) ℓ_i will denote the width of the non-zero portion of the curve, (iii) m_i, ℓ_i remain independent of each other, and (iv) m_i, ℓ_i can both decrease all the way to zero, with parameter γ controlling the shape of the curve. Example values of r_i are displayed in Fig. S1 via the light blue shading, for $m_i = 1$, $\ell_i = 7$, and $C = 7$. Ovulation signs on each day are depicted by the red shaded bars,

while fertility probabilities are given by the black lines.

A.1.3 Process Overview and Scheduling

The model proceeds in discrete, non-overlapping generations. Males and receptive females compete for mating opportunities. Mating is followed by offspring production and dispersal. Males can also engage in infanticide. Within each generation, reproduction occurs based on female fitness, which in turn is based on mating success during the female reproductive cycle. The relative contribution of a female to the offspring generation is proportional to her relative fitness payoff. Hence, "fitness" can be thought of as the expected number of offspring surviving to the age of reproduction, or each female's relative reproductive success. For each trait, mutations occur with a small probability. Females are also able to migrate between groups.

We consider three different sets of results regarding selection:

(1) Evolution of receptivity (r) given fixed concealed ovulation ($m, \ell = 0$),

(2) Evolution of receptivity (r) given fixed ovulation signs present ($m = 0.9, \ell = 4$), and

(3) Evolution of receptivity (r), ovulation signs magnitude (m), and ovulation signs length (ℓ).

A.2 Design Concepts

A.2.1 Emergence

Individual differences in female-expressed traits (ovulation signs magnitude m, ovulation signs length ℓ, and receptivity r) emerge, as individuals that perform poorly are eliminated over time and replaced by individuals adopting the most efficient strategy. The initial gene pool is initiated at random in the first generation.

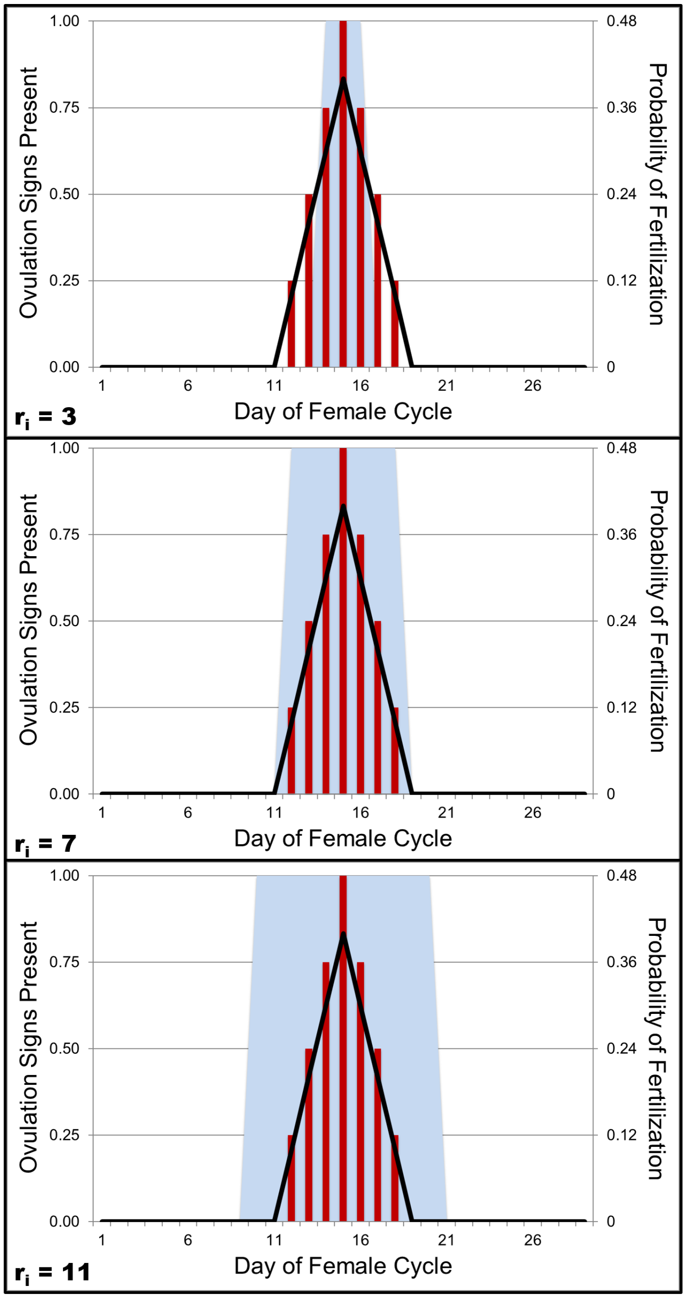

Figure S1: Sample receptivity lengths are displayed for $r_i = 3, 7, 11$ (from top to bottom) via the light blue shading. Each graph also includes ovulation signs present (with $m_i = 1, \ell_i = 7$) via the red bars and fertilization probabilities (with $C = 7$) via the black lines. In the top graph, we see that a female can be fertile and/or have ovulation signs visible on days where she is not receptive. In the bottom graph, we instead see a female can be receptive on days where she does not have any ovulation signs visible and/or is not fertile. The middle graph depicts the situation where visible ovulation signs, fertility, and receptivity all line up with lengths of 7 days.

A.2.2 Adaptation

Each female is characterized by up to three evolving traits. r is the length of time a female is receptive to mating (a non-negative, integer value). In addition, females may also be characterized by their visual ovulation signs, denoted $x(d)$ for each day d of the cycle, thought of as overlapping curves. Visual ovulation signs are characterized by two genetically-controlled traits. Ovulation signs magnitude m is the maximum amount of ovulation signs a female has visible during her cycle (a non-negative, continuous value), while ovulation signs length ℓ is the number of days a female has *some* amount of ovulation signs visible (a non-negative, integer value). The evolution of each of evolving trait is governed by female fitness, or each female's relative reproductive success.

A.2.3 Fitness

The fitness function for female i in the model is as follows:

$$w_i = w_0 + (1-\eta)y_{g,i} + \eta\tilde{y}_{ng} + \sum_{j=1}^{N}\left[f(j)g(p_{i,j})\right] - c\bar{x}_i - c_r\frac{r_i}{D}. \tag{S2}$$

Here, w_0 is baseline fitness, $y_{g,i}$ the genetic benefit provided by the male who fertilizes female i, $\tilde{y}_{ng} = \frac{1}{D}\sum_{(\text{all mates } j)} y_{ng,j}$ the average non-genetic benefit provided by all female i's mates, $p_{i,j}$ the perceived paternity probability of male j for female i's offspring, $c \cdot \bar{x}_i$ the costs to a female of supporting her visual ovulation signs with $\bar{x}_i = \frac{1}{D}\sum_{d=1}^{D} x_i(d)$, a female's average visual ovulation signs, and $c_r\frac{r_i}{D}$ the costs to a female of being receptive to mating for $\frac{r_i}{D}$ proportion of her cycle.

Notice for the case when $\alpha = 0$ and, thus, $g = 0$ (i.e., without the effects of infanticide), the fitness function above collapses into

$$w_i = w_0 + (1-\eta)y_{g,i} + \eta\tilde{y}_{ng} - c\bar{x}_i, -c_r\frac{r_i}{D}. \tag{S3}$$

Note in this $\alpha = 0$ (no infanticide) case, parameters β, κ, τ, and ω all become unnecessary.

A.2.4 Learning and Prediction

Individuals in the model do not change their behavior in response to experience. In addition, individuals in the model do not predict future consequences of their decisions.

A.2.5 Sensing

All sensing is local. Males and females can identify the opposite sex and can discriminate during mate choice. Males will preferentially mate with females with more ovulation signs visible, and females will only mate with males on days when they are receptive. Males will also know which females they mated with during a cycle, in order to "estimate" their probability of paternity.

A.2.6 Interaction

Interaction between individuals occurs through both mating and infanticide. See "Submodels" for more details on infanticide.

We assume each female to mate once on every day on which she is receptive, and each male to be able to mate with no more than one female on any given day. This means if there are R receptive females on any given day of the cycle, there will only be R males able to mate with a female on that day. Biologically, a male after mating with a female may guard her to ensure no other male is able to mate with her (i.e., mate guard) and/or a male must do other activities besides mating/searching for mates (e.g., hunting/eating). Mating is followed by offspring production and dispersal.

We assume that, for every day of the cycle, males of higher GC will preferentially mate with females with more ovulation signs visible. Parameters ϵ_m and ϵ_f control the amount of stochasticity in the process of mating pair formation. On each day, mating pairs are formed by first randomly perturbing both the male trait y_g and female trait x by adding to each an independent, normally distributed random variable with standard deviation $\epsilon_m \geq 0$ and $\epsilon_f \geq 0$, respectively (i.e., $x' = x + e_x, y'_g = y_g + e_y$, where $e_x \sim N(0, \epsilon_f^2), e_y \sim N(0, \epsilon_m^2)$). Males and receptive females are then sorted according to these perturbed values and mating occurs between individuals of

the same order. With $\epsilon_m, \epsilon_f \to \infty$, mating pairs are formed completely independently of the values of x_i and $y_{g,j}$.

A.2.7 Stochasticity

Stochasticity is present in mating, reproduction, and mutation. Stochasticity in mating pairs comes from ϵ_m, ϵ_f, as described above. Stochasticity in reproduction comes from each male mate having some probability of paternity, as described below. Both the occurrence and the effect of mutations are stochastic. The probability of mutation (per gene, per generation) is given by μ. For the continuous trait m, mutations are selected from $N(0, \sigma^2)$. For the integer traits r, ℓ, mutations are instead taken from the set $\{-1, 1\}$ with equal probability.

A.2.8 Collectives

The population and gene pool are the two collective levels. The population is important, since female fitness is impacted by both her male mate(s) and infanticide. The gene pool acts through the model's population of sexually-reproducing individuals.

A.2.9 Observation

In each generation, the evolving female-expressed traits - that is, r, m, and ℓ - of all individuals in the population are recorded.

A.3 Details

A.3.1 Initialization

For this model, recall we consider 3 different sets of simulations regarding selection:

(1) Evolution of receptivity (r) given fixed concealed ovulation ($m, \ell = 0$),

(2) Evolution of receptivity (r) given fixed ovulation signs present ($m = 0.9, \ell = 4$), and

(3) Evolution of receptivity (r), ovulation magnitude (m), and ovulation length (ℓ).

For (1) and (2), we use the following 10 initial conditions: $r = 1, 4, 7, 10, 13, 16, 19, 22, 25, 28$. For (3)'s initial conditions, we use every combination of $r = 3, 11, 19, 27$, $m = 0.3, 0.9$, and $\ell = 2, 4$. Parameters used for all the above simulations include: $G = 400$; $T = 100,000$; $D = 29$; $C = 7$ (with probabilities of fertilization on each of these days being 0.125, 0.25, 0.375, 0.5, 0.375, 0.25, 0.125, respectively); $w_0 = 2$; $N = 4, 8, 16$; $b = 0.1, 0.2, 0.4$; $c = 0.1, 0.2, 0.4$; $c_r = 0.4, 0.6, 0.8$; $\eta = 0.25, 0.5, 0.75$; $\rho = -0.5, 0, 0.5$; $\gamma = 1$; $\hat{y} = 1$; $\sigma = \sqrt{0.1}$; $\epsilon_m = 0, 0.25, 0.5$; $\epsilon_f = 0, 0.01, 0.05$, $\alpha = 0, 0.2, 0.6, 1$; $\beta = 1.25, 1.5, 1.75$; $\tau = 2$; $\omega = 1$; and $\kappa = 0, 0.5, 1$. These are summarized in Table S1.

A.3.2 Input Data

This simulation model does not use input data.

A.3.3 Submodels

There are two submodels considered in the overall model. The first is calculating probabilities of paternity for all males, and the second is infanticide by males.

To calculate the probability of paternity for each male, first the fertility of each female with which that male has mated is summed up for every day a particular male mates with her. These values are normalized across males for each female in the group, meaning a male who mates with the female on a day where she has a higher fertility probability will have a higher paternity probability. The actual father for that female's offspring is then determined randomly, proportional to each male's probability of paternity for her offspring.

Note these *actual* probabilities of paternity are different from the *perceived* probabilities of paternity, as detailed in the main text. We make this distinction because males will not know any female's probability of fertility at the time he mates with her; a male will only know how many visible ovulation signs she has present. Separating these quantities allows *actual* probabilities of paternity (which males do not know) to be calculated using females' probabilities of fertility, and *perceived* probabilities of paternity (which males *do* know) to be calculated using females' visible

ovulation signs.

In addition, each male in a female's group can affect her fitness directly, due to infanticide, although males will differ in their actions' effectiveness (e.g., due to strength, rank, size, alliances, etc.). We assume each male's effectiveness to be proportional to $f(j)$ for each male j (sorted by males' y_g) via an exponential function: $f(j) \sim e^{-\omega j}$ with parameter $\omega > 0$ controlling the amount of disparity among males in their corresponding effectiveness within the group. Note a larger value of ω indicates more disparity, and $\omega = 0$ equality. We define this exponential function of male effectiveness to be:

$$f(j) = \frac{e^{-\omega j}}{\sum_{k=1}^{N} e^{-\omega k}}. \tag{S4}$$

A male's contribution $g(p_j)$ to female fitness (positive via helping protect the offspring, or negative via not helping and/or harming the offspring) depends on his perceived probability of paternity p_j. We define $g(p_j) = \alpha[1 - \beta(1 - p_j)^\tau]$, with parameters $\alpha, \beta > 0$ and $\tau \geq 1$. Note α determines the maximum benefit a female can obtain from a male protecting her offspring from infanticide, while $\alpha(1 - \beta)$ determines the maximum cost a female can incur from a male *not* protecting her offspring from infanticide. The overall effect of infanticide on female fitness is $\sum_j f(j)g(p_j)$, summing over all males j. Note whenever $\alpha = 0$, there are no effects of infanticide.

A male in general would not know his actual paternity probability and would instead have to estimate it, when decided on protecting an infant or committing infanticide. Consider male j mating with female i with visible ovulation signs $x_i(d)$. We postulate that the male-estimated ("perceived") probability p_j of being the father of her offspring is proportional to $(x_i(d))^\kappa$ where $\kappa \in [0, 1]$ is the weight males put on visual ovulation signs. With multiple matings with the same female, we sum up the corresponding terms $(x_i(d))^\kappa$.

B List of all Parameters

Parameters used in the model are outlined in Table S1. For each parameter, one value listed implies the parameter remained fixed throughout all simulations, and multiple values indicates

each of those parameter values was tested in all combinations with other parameters.

Table S1: List of All Parameters

**Parameter Name**	**Parameter Description**
$G = 400$	Number of groups
$T = 100000$	Time (number of generations)
$D = 29$	Number of days in each female's cycle
$C = 7$	Number of days each female has non-zero probability of fertilization
$C_v = \{0.125, 0.25, 0.375, 0.5, 0.375, 0.25, 0.125\}$	Probability of fertilization on each of those C = 7 days
$w_0 = 2$	Baseline female fitness
$N = 4, 8, 16$	Number of males and number of females in each group
$b = 0.1, 0.2, 0.4$	Variation in male quality
$c = 0.1, 0.2, 0.4$	Cost of having ovulation signs present
$c_r = 0.4, 0.6, 0.8$	Cost of being receptive to mating
$\eta = 0.1, 0.2, 0.4$	Weighting of NGC vs. GC on a female's fitness
$\rho = -0.5, 0, 0.5$	Correlation between a male's GC and NGC
$\hat{y} = 1$	Mean male genetic quality
$\epsilon_m = 0, 0.25, 0.5$	Parameter determining the amount of reproductive stochasticity among males
$\epsilon_f = 0, 0.01, 0.05$	Parameter determining the amount of reproductive stochasticity among females
$\gamma = 1$	Parameter determining the shape of the function for the amount of visual ovulation signs present
$\alpha = 0, 0.2, 0.6, 1$	Parameter controlling the maximum benefit of protecting against infanticide (note α = 0 corresponds to the case with NO infanticide)
$\beta = 1.25, 1.5, 1.75$	Parameter controlling the relative maximum cost of infanticide in the paternity probability effect function
$\tau = 2$	Exponent in the paternity probability effect function g
$\omega = 1$	Parameter in the male effectiveness function $f(j)$
$\kappa = 0, 0.5, 1$	Parameter determining the weight males put on the amount of visual ovulation signs in their paternity estimate
$\nu = 1$	Migration rate (between zero and one)
$\mu = 0.001$	Probability of mutation (per gene per generation)
$\sigma = \sqrt{0.1}$	Standard deviation for mutation magnitude

C Effects of all Parameters

See Table S2 for a summary of effects for all main parameters used in the model. These effects are all illustrated for simulations with concealed ovulation fixed (Figures S2,S3,S4), visible ovulation signs fixed (Figures S5,S6,S7), and each of receptivity length, ovulation signs magnitude, and ovulation signs length evolving (Figures S8-S16).

Table S2: Effects of All Parameters

Traits Evolving:	Found in Figure:	Value(s) of ρ:	Other Parameters Investigated:
Only receptivity r, with concealed ovulation fixed ($m = 0, \ell = 0$)	S2	$\rho = 0.5, 0, -0.5$	N, b, c_r, η
	S3	$\rho = 0.5, 0, -0.5$	$\alpha, \beta, \kappa, \eta$
	S4	$\rho = 0.5, 0, -0.5$	$\epsilon_m, \epsilon_f, c_r, \eta$
Only receptivity r, with fixed visual ovulation signs ($m = 0.9, \ell = 4$)	S5	$\rho = 0.5, 0, -0.5$	N, b, c_r, η
	S6	$\rho = 0.5, 0, -0.5$	$\alpha, \beta, \kappa, \eta$
	S7	$\rho = 0.5, 0, -0.5$	$\epsilon_m, \epsilon_f, c_r, \eta$
Receptivity length r, ovulation signs magnitude m, and ovulation signs length ℓ	S8	$\rho = 0.5$	N, b, c, c_r, η
	S9	$\rho = 0$	N, b, c, c_r, η
	S10	$\rho = -0.5$	N, b, c, c_r, η
	S11	$\rho = 0.5$	$\alpha, \beta, \kappa, \eta$
	S12	$\rho = 0$	$\alpha, \beta, \kappa, \eta$
	S13	$\rho = -0.5$	$\alpha, \beta, \kappa, \eta$
	S14	$\rho = 0.5$	$\epsilon_m, \epsilon_f, c_r, \eta$
	S15	$\rho = 0$	$\epsilon_m, \epsilon_f, c_r, \eta$
	S16	$\rho = -0.5$	$\epsilon_m, \epsilon_f, c_r, \eta$

C.1 ANOVA Results

We ran an analysis of variance (ANOVA) to determine which of receptivity length (r), ovulation signs magnitude m, and ovulation signs length ℓ are affected most by which parameters, as introduced in the main text.

The following tables (Tables S3, S4, S5) give the detailed results of these tests. Each table below reflects the results from different simulation sets. The numbers in each of these tables correspond to percentage of variance, with the sign ($\pm$) corresponding to the direction of the effect. Any table entry with a zero means that effect is not significant (i.e., $p > 0.05$). For example, in Table S3, 31% of the variation in receptivity length (r) in this simulation set can be explained by parameter c_r, and another 38% by parameter η. Since the effect of c_r is negative and η positive, we know that as c_r increases, r decreases, and as η increases, r instead increases. Each of the following tables can be interpreted in this fashion.

Table S3: Effects of N, b, c, c_r, η, ρ on receptivity length r and ovulation signs magnitude m and length ℓ

	r	m	ℓ
N	0	0.019	0
b	0	0.261	0.179
c	0	-0.171	-0.313
c_r	-0.313	-0.072	-0.124
η	0.380	0.005	0.105
ρ	0	0.014	0.029
error	0.306	0.459	0.530

Table S4: Effects of $\alpha, \beta, \kappa, \eta, \rho$ on receptivity length r and ovulation signs magnitude m and length ℓ

	r	m	ℓ
α	0.025	0.326	0.187
β	0	0.021	0.007
κ	-0.132	0.422	-0.294
η	0.634	-0.022	0.270
ρ	0.0002	0	0.020
error	0.207	0.209	0.222

Table S5: Effects of $\epsilon_m, \epsilon_f, c_r, \eta, \rho$ on receptivity length r and ovulation signs magnitude m and length ℓ

	r	m	ℓ
ϵ_m	0	-0.148	-0.017
ϵ_f	0	-0.011	-0.118
c_r	-0.317	-0.151	-0.122
η	0.375	0	0.117
ρ	0	0.021	0.034
error	0.308	0.661	0.593

C.2 Supplementary Figures

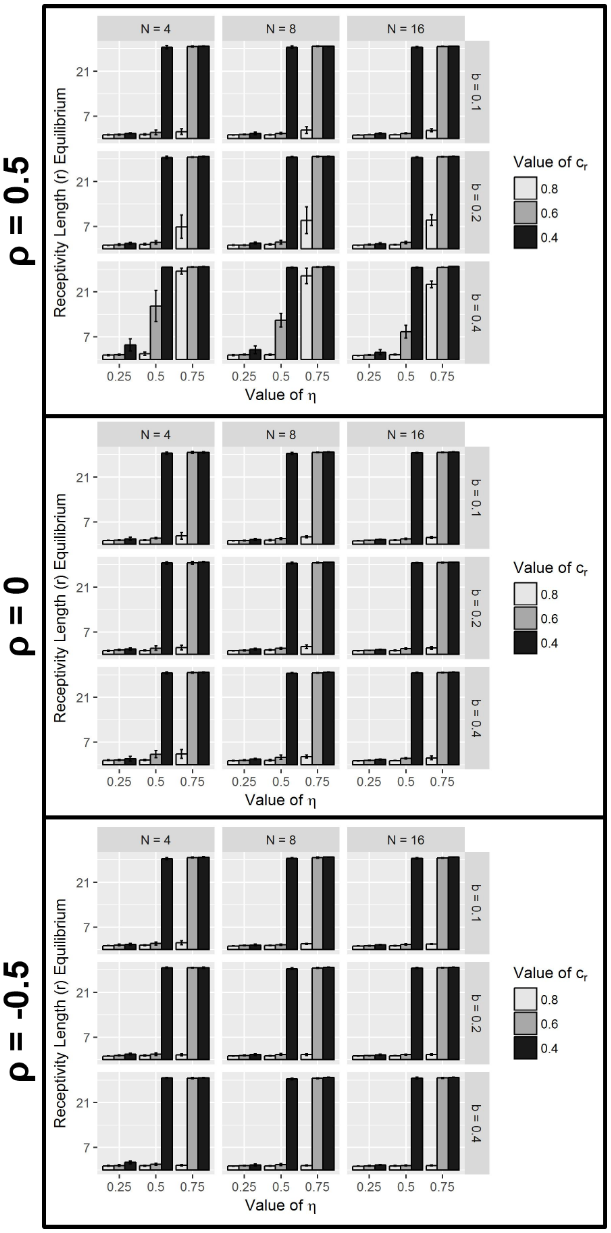

Figure S2: The effects of parameters N (group size), b (benefit of male genetic quality), c_r (cost of receptivity length), and η (relative weighting of NGC) on the average equilibria values of receptivity length (r) for three different values of ρ (correlation between males' GC and NGC), when concealed ovulation is fixed ($m = 0, \ell = 0$) for all females. Equilibria are obtained by averaging over 10 initial condition runs (with standard deviation indicated by error bars). All other parameters were held constant: $\alpha = 0, c = 0.2, \epsilon_m = 0.25, \epsilon_f = 0.01$.

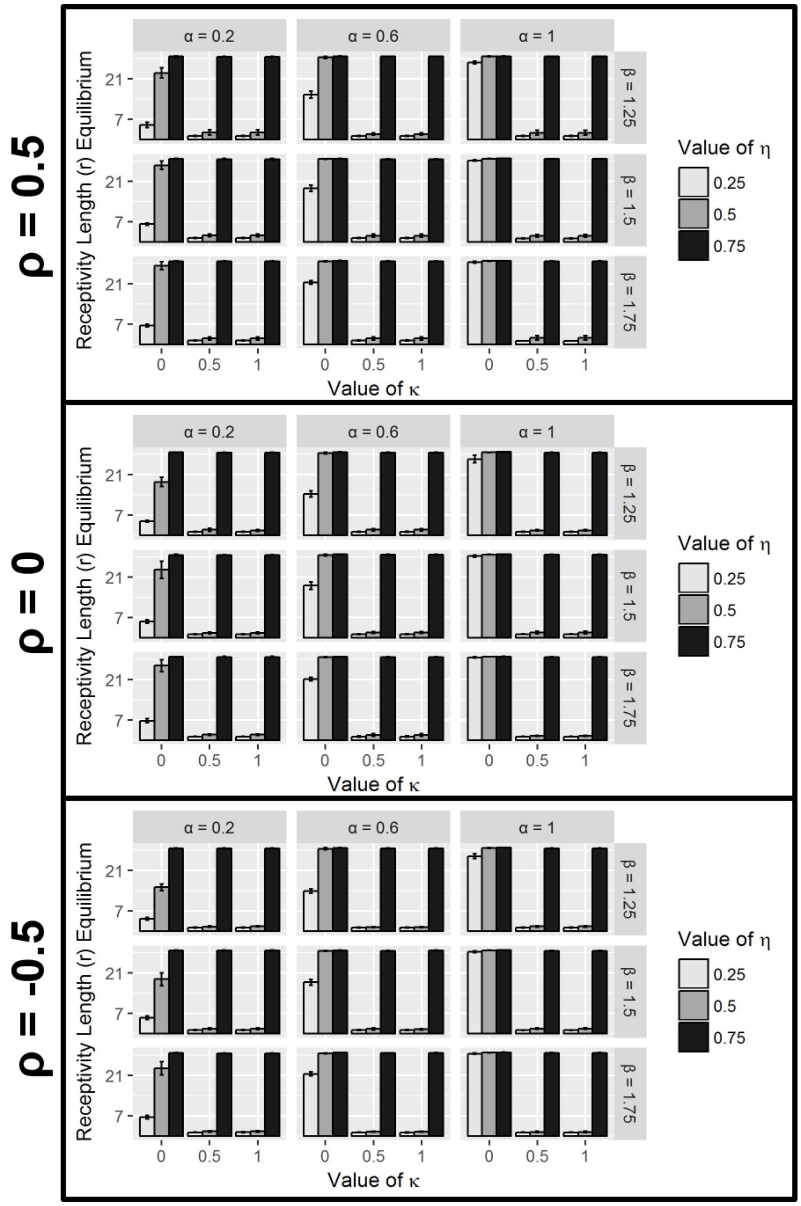

Figure S3: The effects of parameters α (maximum benefit of protecting offspring from infanticide), β (maximum cost of not protecting offspring from infanticide), κ (weight males put on visible ovulation signs when estimating their paternity), and η (relative weighting of NGC) on the average equilibria values of receptivity length (r) for three different values of ρ (correlation between males' GC and NGC), when concealed ovulation is fixed ($m = 0, \ell = 0$) for all females. Equilibria are obtained by averaging over 10 initial condition runs (with standard deviation indicated by error bars). All other parameters were held constant: $N = 8, b = 0.2, c = 0.2, c_r = 0.6, \epsilon_m = 0.25, \epsilon_f = 0.01$.

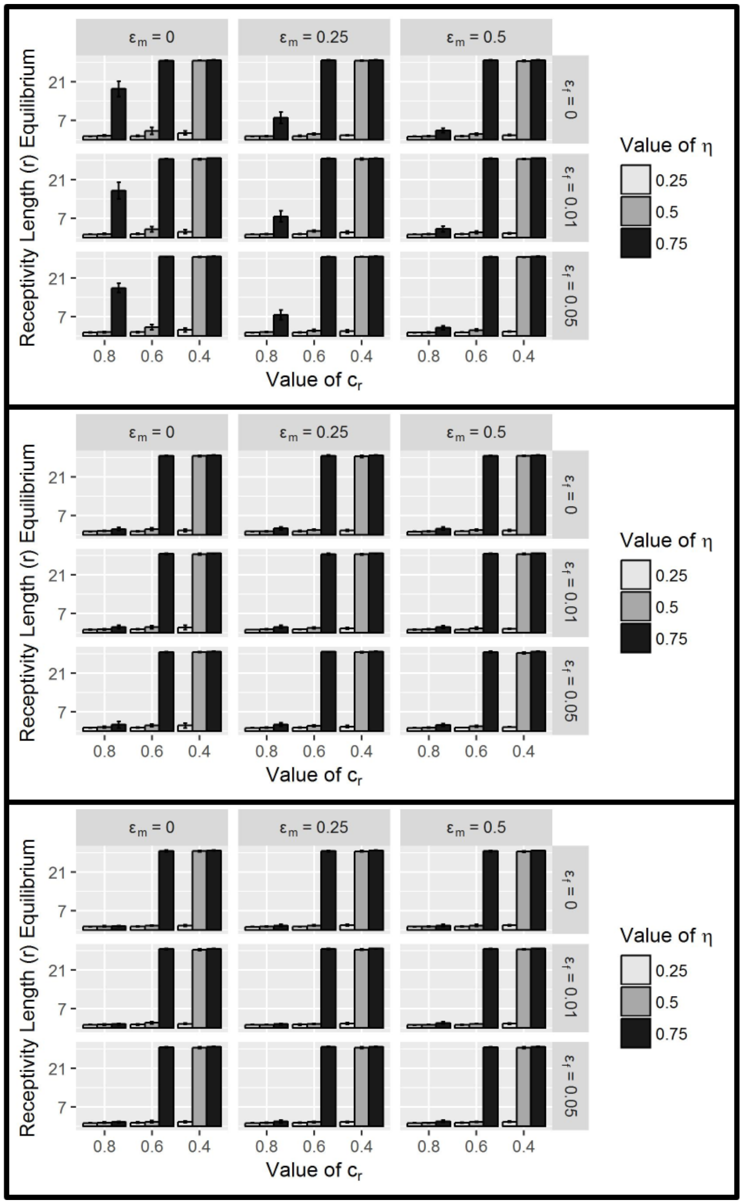

Figure S4: The effects of parameters ϵ_m (male reproductive stochasticity), ϵ_f (female reproductive stochasticity), c_r (cost of receptivity length), and η (relative weighting of NGC) on the average equilibria values of receptivity length (r) for three different values of ρ (correlation between males' GC and NGC), when concealed ovulation is fixed ($m = 0, \ell = 0$) for all females. Equilibria are obtained by averaging over 10 initial condition runs (with standard deviation indicated by error bars). All other parameters were held constant: $\alpha = 0, N = 8, b = 0.2, c = 0.2$.

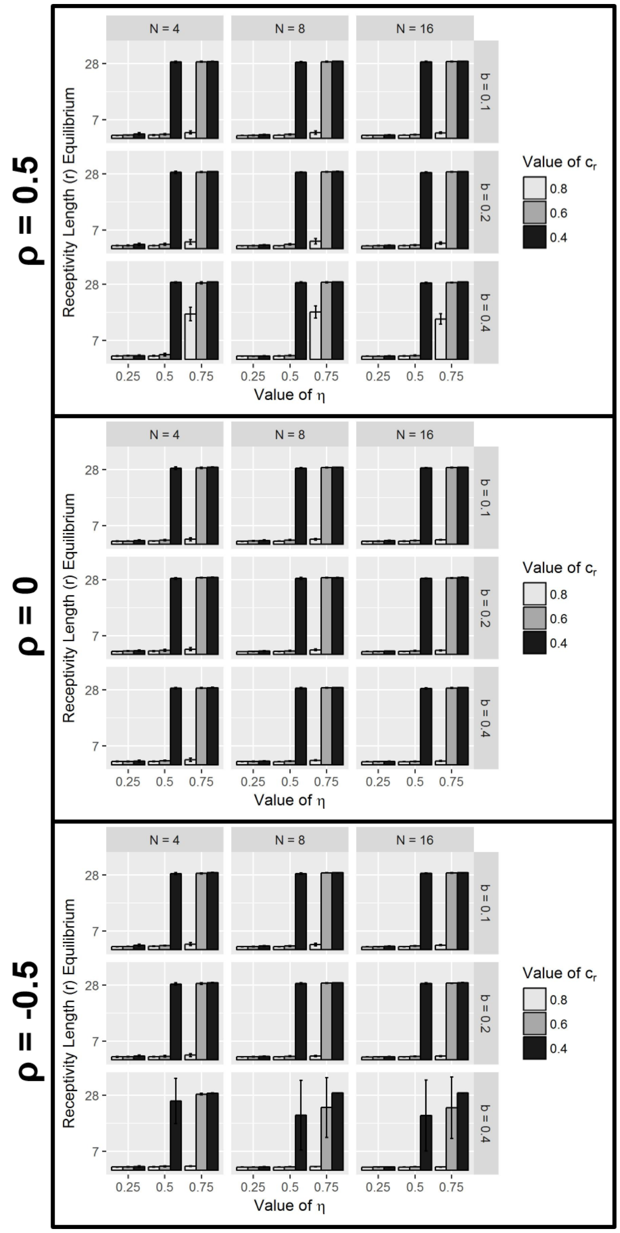

Figure S5: The effects of parameters N (group size), b (benefit of male genetic quality), c_r (cost of receptivity length), and η (relative weighting of NGC) on the average equilibria values of receptivity length (r) for three different values of ρ (correlation between males' GC and NGC), when visible ovulation signs are fixed ($m = 0.9, \ell = 4$) for all females. Equilibria are obtained by averaging over 10 initial condition runs (with standard deviation indicated by error bars). All other parameters were held constant: $\alpha = 0, c = 0.2, \epsilon_m = 0.25, \epsilon_f = 0.01$.

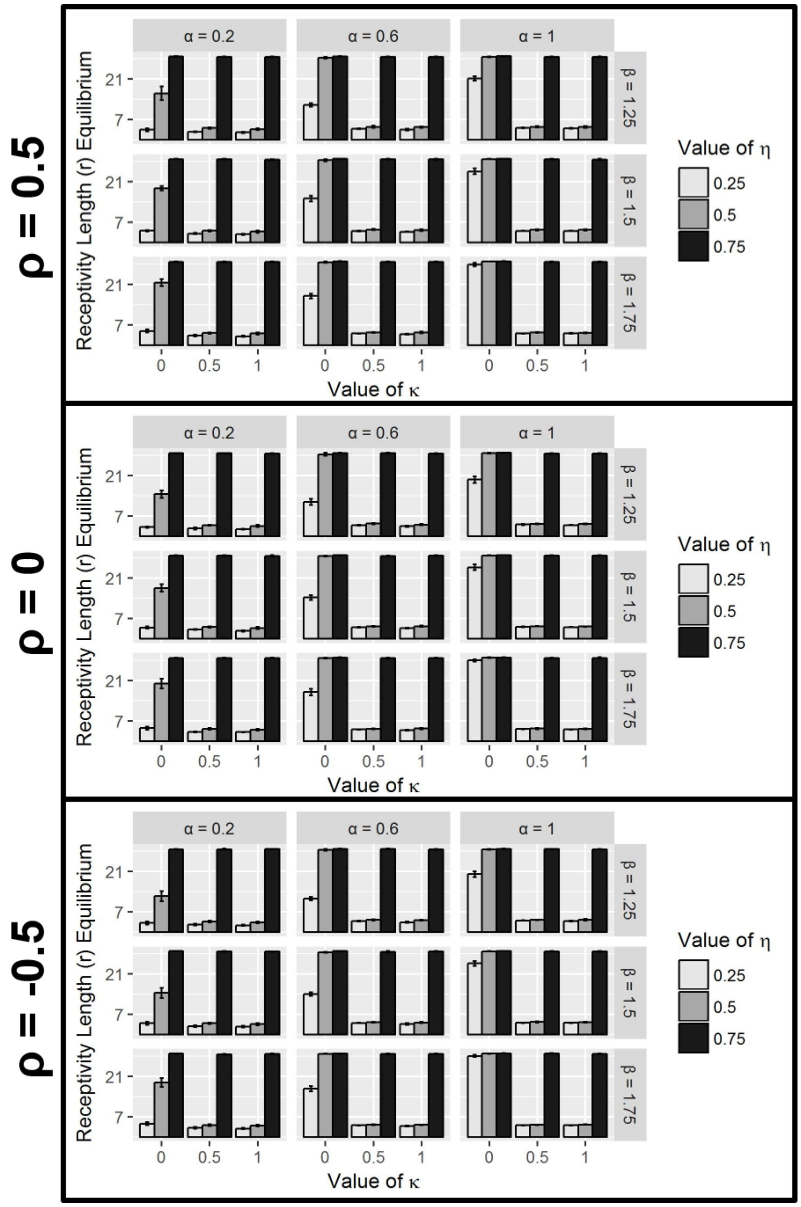

Figure S6: The effects of parameters α (maximum benefit of protecting offspring from infanticide), β (maximum cost of not protecting offspring from infanticide), κ (weight males put on visible ovulation signs when estimating their paternity), and η (relative weighting of NGC) on the average equilibria values of receptivity length (r) for three different values of ρ (correlation between males' GC and NGC), when visible ovulation signs are fixed ($m = 0.9, \ell = 4$) for all females. Equilibria are obtained by averaging over 10 initial condition runs (with standard deviation indicated by error bars). All other parameters were held constant: $N = 8, b = 0.2, c = 0.2, c_r = 0.6, \epsilon_m = 0.25, \epsilon_f = 0.01$.

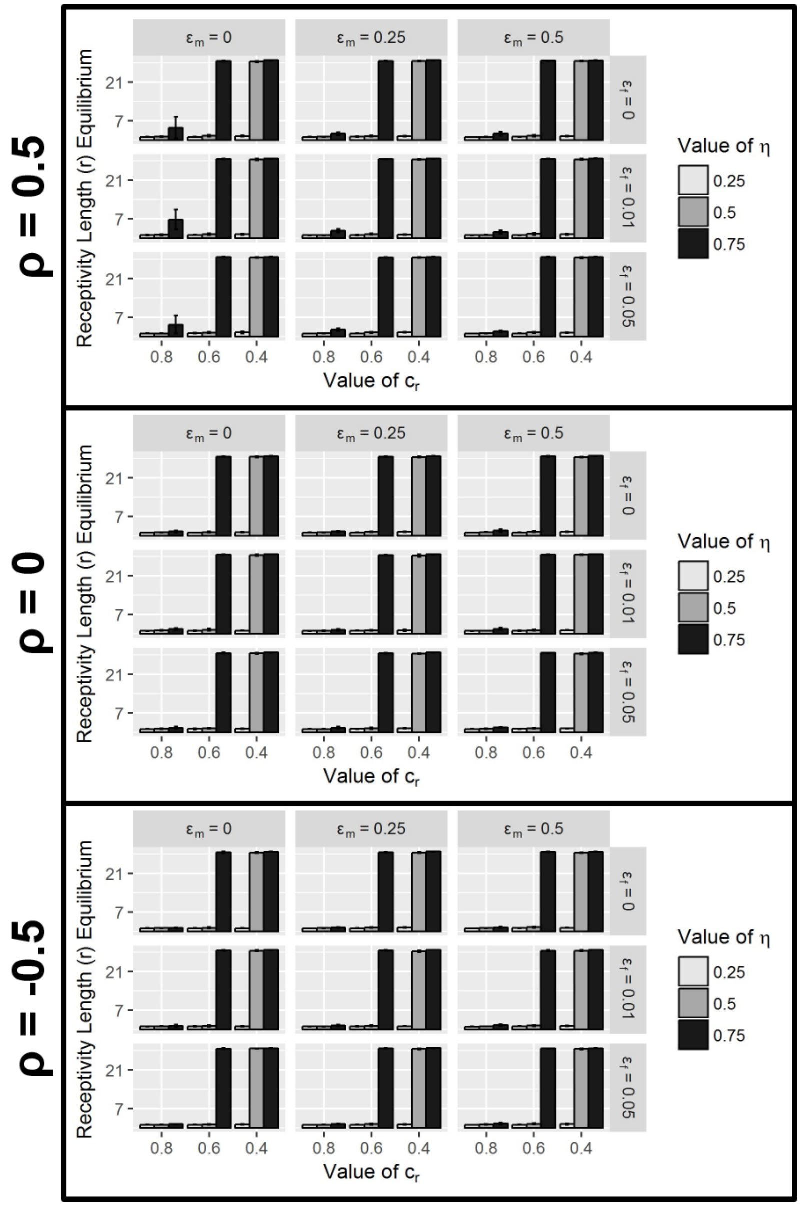

Figure S7: The effects of parameters ϵ_m (male reproductive stochasticity), ϵ_f (female reproductive stochasticity), c_r (cost of receptivity length), and η (relative weighting of NGC) on the average equilibria values of receptivity length (r) for three different values of ρ (correlation between males' GC and NGC), when visible ovulation signs are fixed ($m = 0.9, \ell = 4$) for all females. Equilibria are obtained by averaging over 10 initial condition runs (with standard deviation indicated by error bars). All other parameters were held constant: $\alpha = 0, N = 8, b = 0.2, c = 0.2$.

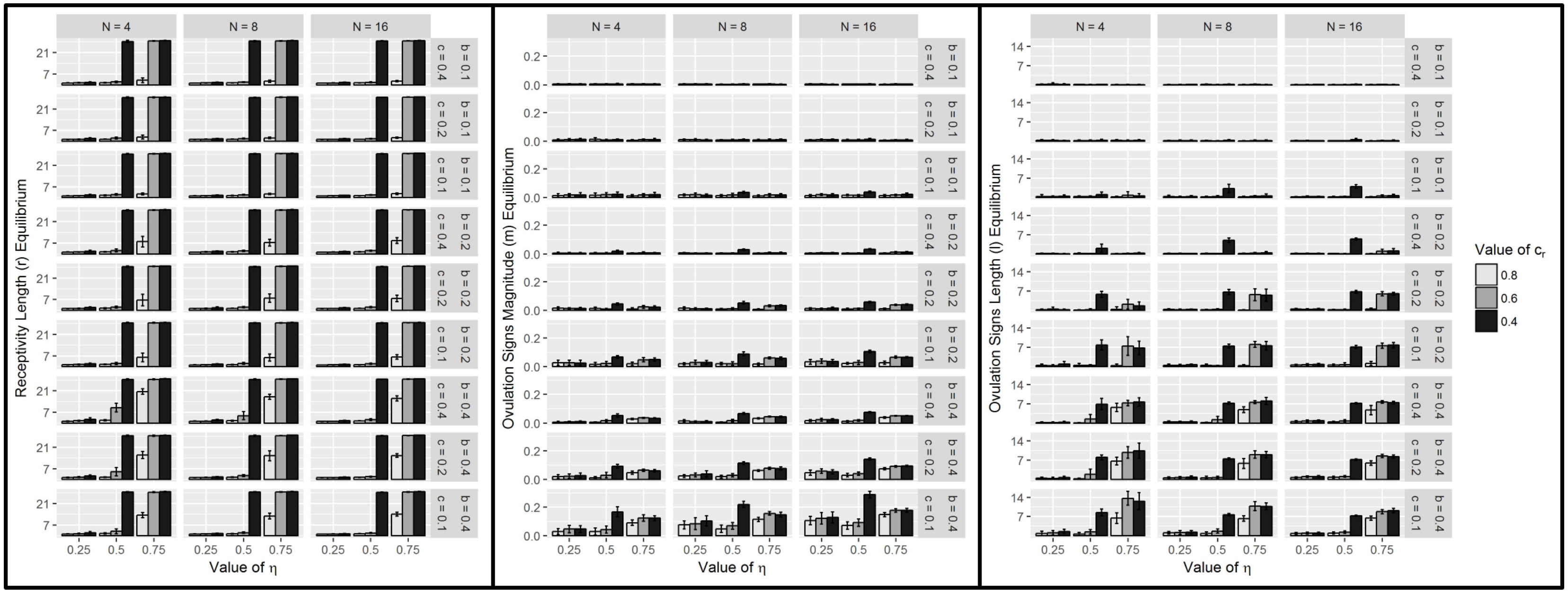

Figure S8: The effects of parameters N (group size), b (benefit of male genetic quality), c (cost of visible ovulation signs), c_r (cost of receptivity length), and η (relative weighting of NGC) on the average equilibria values of receptivity length (r), ovulation signs magnitude (m), and ovulation signs length (ℓ) with $\rho = 0.5$ (correlation between males' GC and NGC). Equilibria are obtained by averaging over 16 initial condition runs (with standard deviation indicated by error bars). All other parameters were held constant: $\alpha = 0, \epsilon_m = 0.25, \epsilon_f = 0.01$.

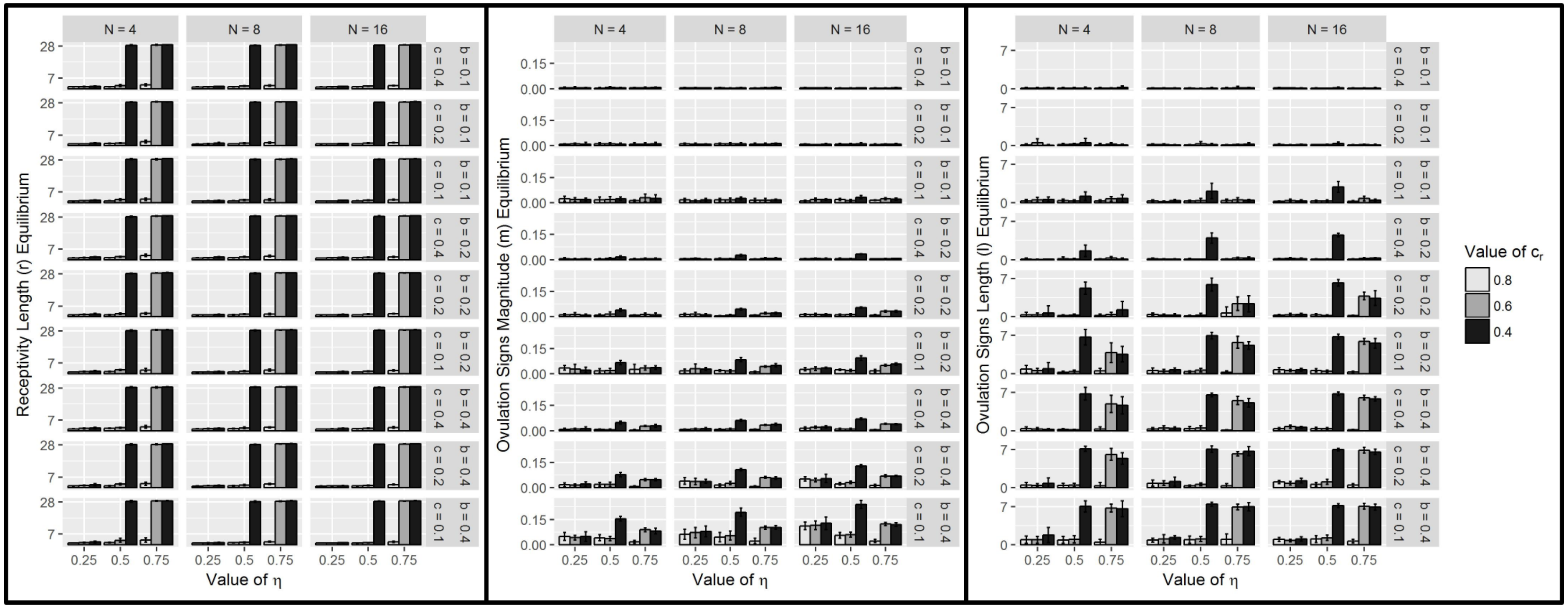

Figure S9: The effects of parameters N (group size), b (benefit of male genetic quality), c (cost of visible ovulation signs), c_r (cost of receptivity length), and η (relative weighting of NGC) on the average equilibria values of receptivity length (r), ovulation signs magnitude (m), and ovulation signs length (ℓ) with $\rho = 0$ (correlation between males' GC and NGC). Equilibria are obtained by averaging over 16 initial condition runs (with standard deviation indicated by error bars). All other parameters were held constant: $\alpha = 0, \epsilon_m = 0.25, \epsilon_f = 0.01$.

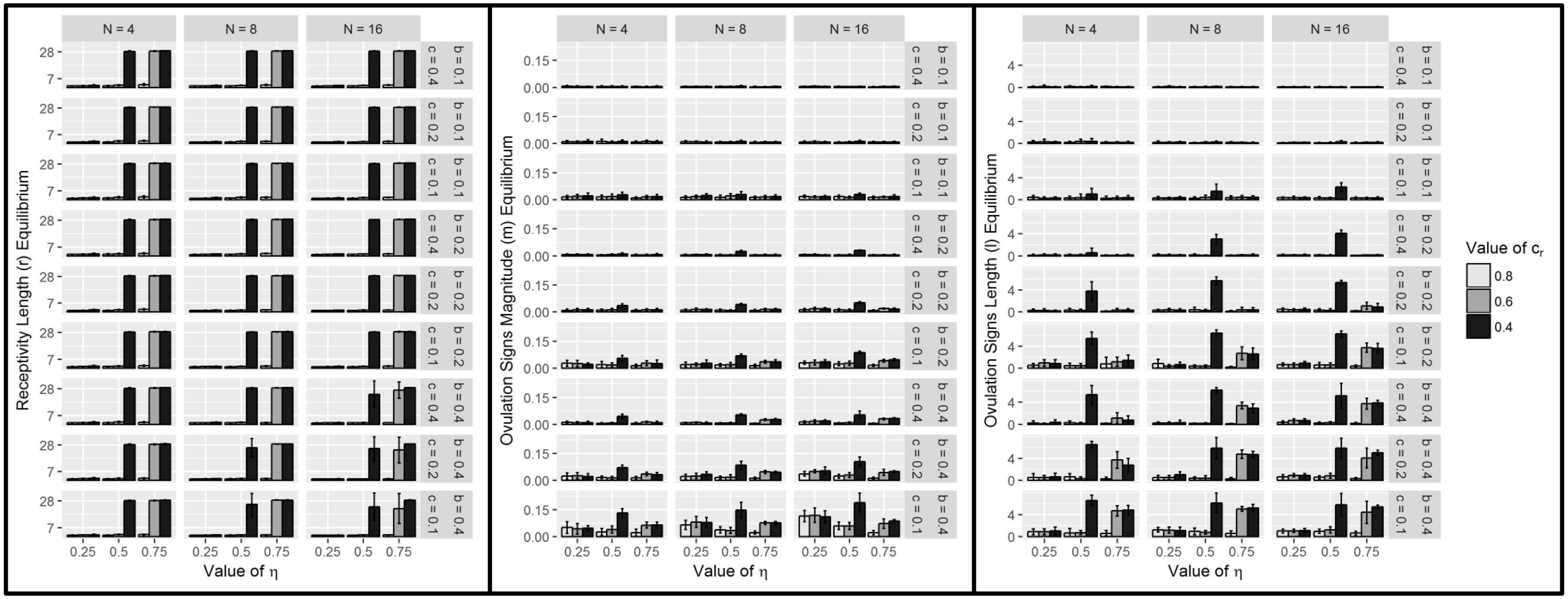

Figure S10: The effects of parameters N (group size), b (benefit of male genetic quality), c (cost of visible ovulation signs), c_r (cost of receptivity length), and η (relative weighting of NGC) on the average equilibria values of receptivity length (r), ovulation signs magnitude (m), and ovulation signs length (ℓ) with $\rho = -0.5$ (correlation between males' GC and NGC). Equilibria are obtained by averaging over 16 initial condition runs (with standard deviation indicated by error bars). All other parameters were held constant: $\alpha = 0, \epsilon_m = 0.25, \epsilon_f = 0.01$.

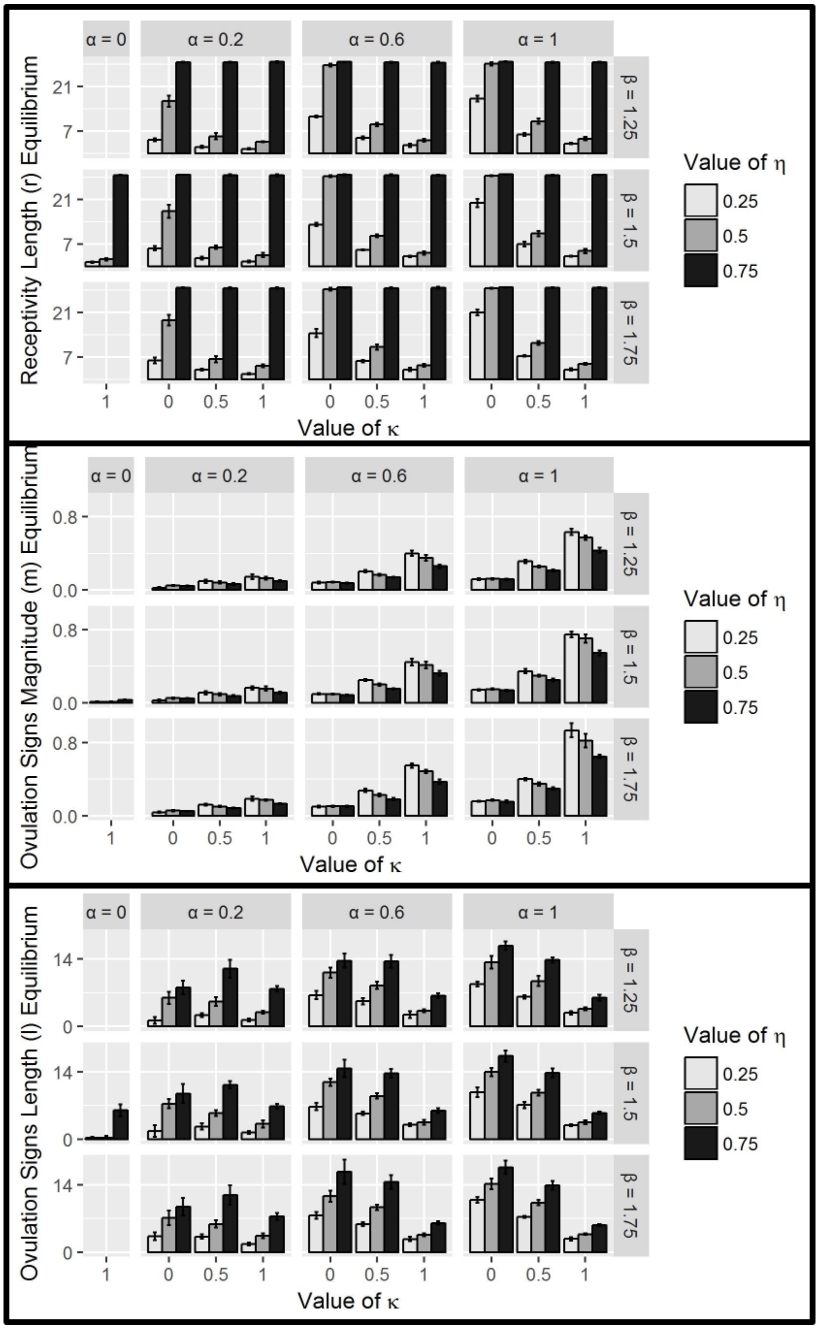

Figure S11: The effects of parameters α (maximum benefit of infanticide), β (proportional to the maximum cost of infanticide), κ (weight males put on females having ovulation signs visible), and η (relative weighting of NGC) on the average equilibria values of receptivity length (r), ovulation signs magnitude (m), and ovulation signs length (ℓ) with $\rho = 0.5$ (correlation between males' GC and NGC). Equilibria are obtained by averaging over 16 initial condition runs (with standard deviation indicated by error bars). All other parameters were held constant: $N = 8, b = 0.2, c = 0.2, c_r = 0.6, \epsilon_m = 0.25, \epsilon_f = 0.01$.

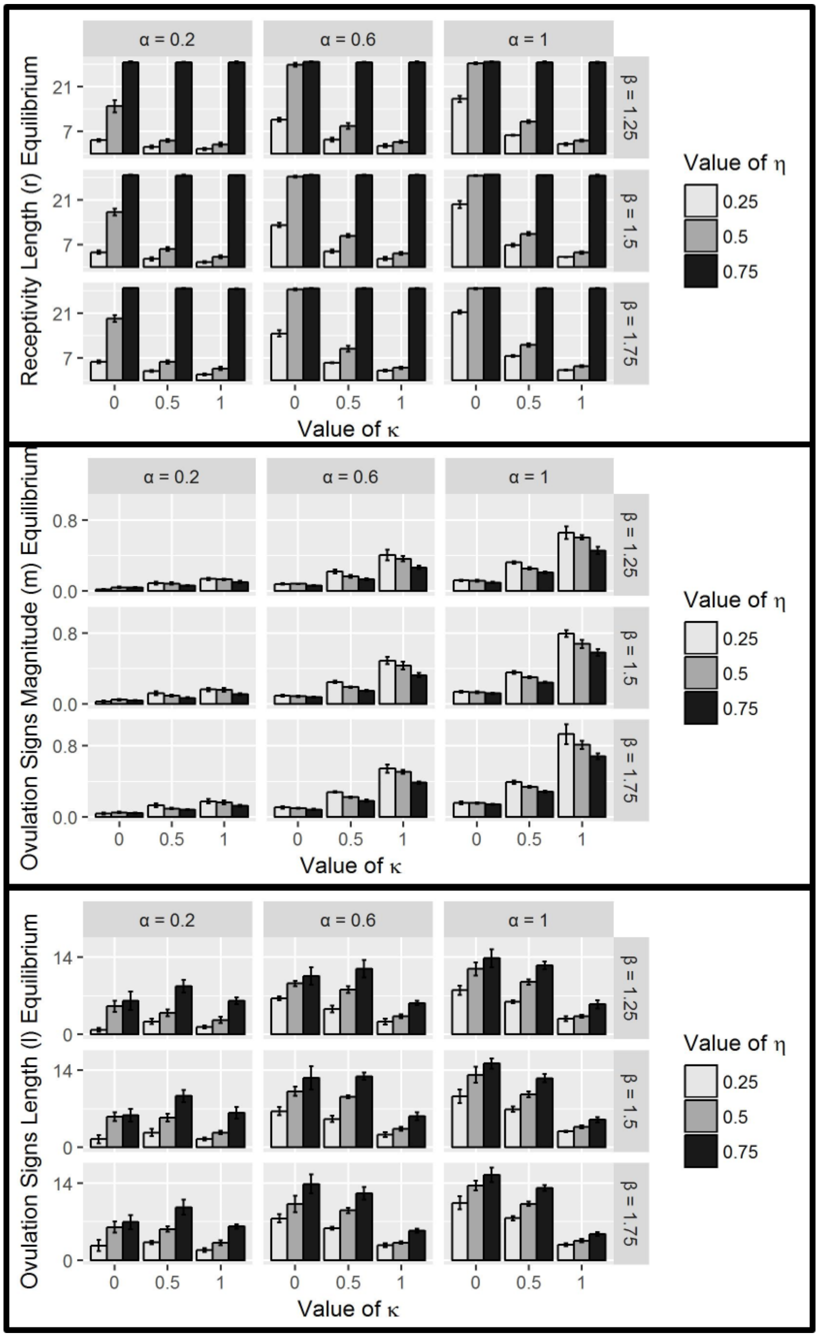

Figure S12: The effects of parameters α (maximum benefit of infanticide), β (proportional to the maximum cost of infanticide), κ (weight males put on females having ovulation signs visible), and η (relative weighting of NGC) on the average equilibria values of receptivity length (r), ovulation signs magnitude (m), and ovulation signs length (ℓ) with $\rho = 0$ (correlation between males' GC and NGC). Equilibria are obtained by averaging over 16 initial condition runs (with standard deviation indicated by error bars). All other parameters were held constant: $N = 8, b = 0.2, c = 0.2, c_r = 0.6, \epsilon_m = 0.25, \epsilon_f = 0.01$.

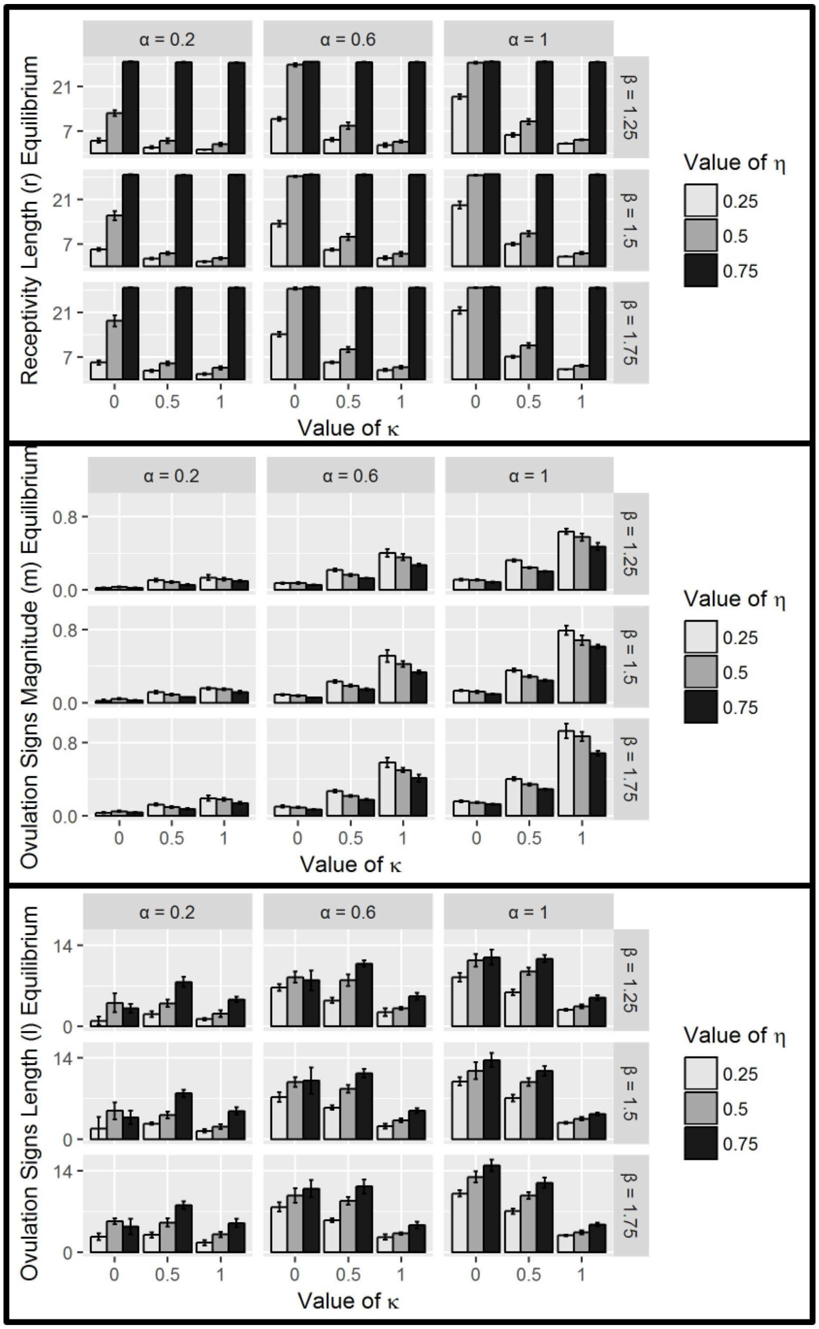

Figure S13: The effects of parameters α (maximum benefit of infanticide), β (proportional to the maximum cost of infanticide), κ (weight males put on females having ovulation signs visible), and η (relative weighting of NGC) on the average equilibria values of receptivity length (r), ovulation signs magnitude (m), and ovulation signs length (ℓ) with $\rho = -0.5$ (correlation between males' GC and NGC). Equilibria are obtained by averaging over 16 initial condition runs (with standard deviation indicated by error bars). All other parameters were held constant: $N = 8, b = 0.2, c = 0.2, c_r = 0.6, \epsilon_m = 0.25, \epsilon_f = 0.01$.

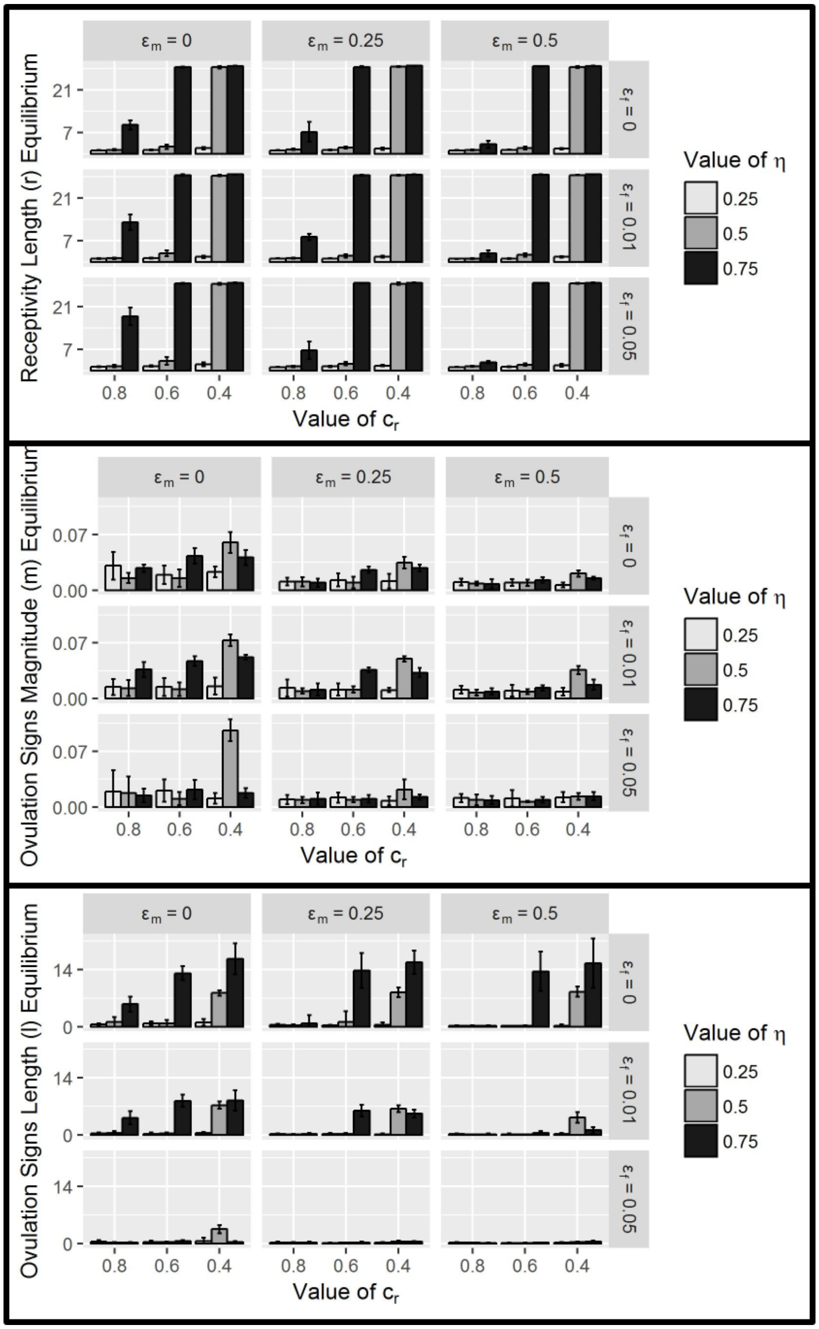

Figure S14: The effects of parameters ϵ_m (reproductive stochasticity among males), ϵ_f (reproductive stochasticity among females), c_r (cost of receptivity length), and η (relative weighting of NGC) on the average equilibria values of receptivity length (r), ovulation signs magnitude (m), and ovulation signs length (ℓ) with $\rho = 0.5$ (correlation between males' GC and NGC). Equilibria are obtained by averaging over 16 initial condition runs (with standard deviation indicated by error bars). All other parameters were held constant: $N = 8, b = 0.2, c = 0.2, \alpha = 0$.

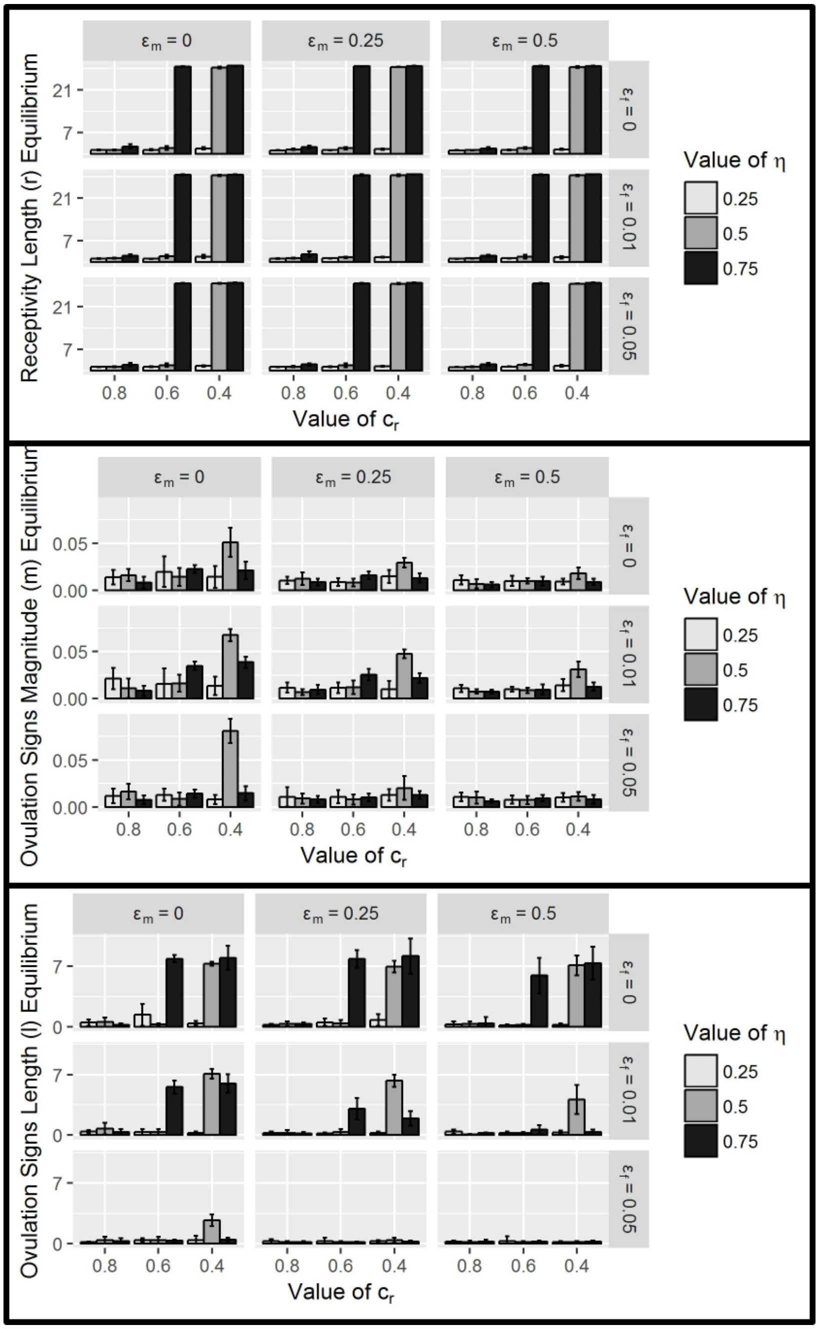

Figure S15: The effects of parameters ϵ_m (reproductive stochasticity among males), ϵ_f (reproductive stochasticity among females), c_r (cost of receptivity length), and η (relative weighting of NGC) on the average equilibria values of receptivity length (r), ovulation signs magnitude (m), and ovulation signs length (ℓ) with $\rho = 0$ (correlation between males' GC and NGC). Equilibria are obtained by averaging over 16 initial condition runs (with standard deviation indicated by error bars). All other parameters were held constant: $N = 8, b = 0.2, c = 0.2, \alpha = 0$.

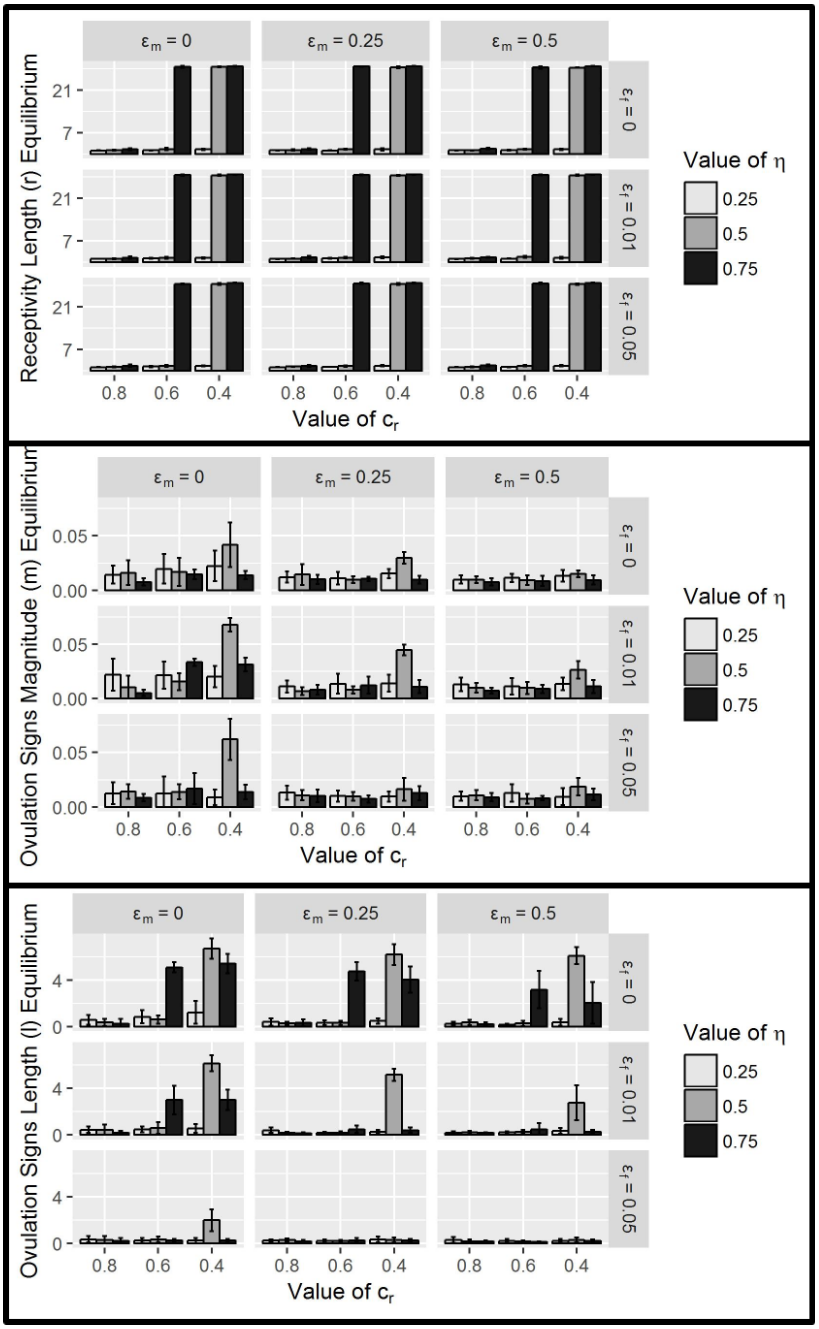

Figure S16: The effects of parameters ϵ_m (reproductive stochasticity among males), ϵ_f (reproductive stochasticity among females), c_r (cost of receptivity length), and η (relative weighting of NGC) on the average equilibria values of receptivity length (r), ovulation signs magnitude (m), and ovulation signs length (ℓ) with $\rho = -0.5$ (correlation between males' GC and NGC). Equilibria are obtained by averaging over 16 initial condition runs (with standard deviation indicated by error bars). All other parameters were held constant: $N = 8, b = 0.2, c = 0.2, \alpha = 0$.
